# Quantum Hall–based superconducting interference device

**DOI:** 10.1126/sciadv.aaw8693

**Published:** 2019-09-13

**Authors:** Andrew Seredinski, Anne W. Draelos, Ethan G. Arnault, Ming-Tso Wei, Hengming Li, Tate Fleming, Kenji Watanabe, Takashi Taniguchi, François Amet, Gleb Finkelstein

**Affiliations:** 1 Department of Physics, Duke University, Durham, NC 27708, USA.; 2 Department of Physics and Astronomy, Appalachian State University, Boone, NC 28607, USA.; 3 Advanced Materials Laboratory, NIMS, Tsukuba 305-0044, Japan.

## Abstract

We present a study of a graphene-based Josephson junction with dedicated side gates carved from the same sheet of graphene as the junction itself. These side gates are highly efficient and allow us to modulate carrier density along either edge of the junction in a wide range. In particular, in magnetic fields in the 1- to 2-T range, we are able to populate the next Landau level, resulting in Hall plateaus with conductance that differs from the bulk filling factor. When counter-propagating quantum Hall edge states are introduced along either edge, we observe a supercurrent localized along that edge of the junction. Here, we study these supercurrents as a function of magnetic field and carrier density.

## Teaser

Pushing superconductivity to the edge: Physicists make a SQUID using quantum Hall edge states.

## INTRODUCTION

The interplay of spin-helical states and superconductivity is predicted to enable access to non-Abelian excitations such as Majorana zero modes (MZM) (*1*–*4*). Through braiding operations that reveal nontrivial exchange statistics, these states may form the basis for quantum computing architectures that take advantage of topological protections to achieve fault tolerance (*5*). Several technologies to this end are in development, including hybrid superconductor-semiconducting nanowire and superconductor-topological insulator structures (*6*, *7*). Interest in topological superconductivity has also spurred a recent flurry of activity at the interface of superconductivity and the quantum Hall (QH) effect (*8*–*21*). It has been predicted that quasi–one-dimensional (1D) superconducting contacts to a QH structure could enable MZM and parafermions (*22*–*25*).

Heterostructures of graphene and hexagonal boron nitride (BN) with 1D superconducting contacts (*10*) can demonstrate a remarkable contact transparency, allowing us to observe supercurrent in the QH regime (*11*). However, the microscopic details of the supercurrent in the QH regime remain an open subject (*17*). In particular, the nature of the superconducting coupling to the edge states could depend, e.g., on the vacuum edges of the graphene mesa, the drift velocity of the QH edge states, or the presence of incompressible strips. Yet, the electrostatic potential along the mesa edge is typically poorly controlled; it is known to be influenced by charge accumulation effects (*26*, *27*) and may be strongly affected by the disorder resulting from physical etching. Here, we examine a graphene Josephson junction with two side gates that allow us to directly manipulate the QH edge states. By tuning either gate, we can change the Landau level filling factor along the edges in a wide range. We controllably induce counter-propagating states along either edge and observe a supercurrent localized solely along one edge. Our measurements unequivocally demonstrate that the supercurrent is carried by the counter-propagating QH edge channels, which are induced by the electrostatic fields at the edges of the sample.

**Fig. 1:**
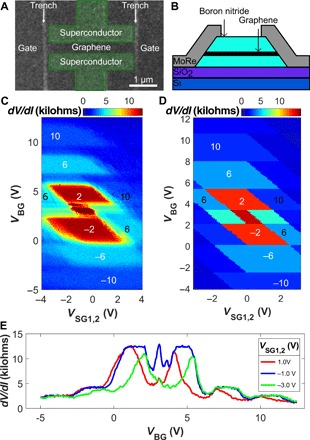
Device layout and gate influence on QH plateaus. (**A**) Scanning electron microscopy (SEM) micrograph of the device prior to reactive ion etching. MoRe contacts are outlined and colored green for contrast. Two trenches (light gray), ~60 nm wide, separate the junction from the side gates. The MoRe contacts are spaced from the trenches by ~100-nm-wide regions of graphene, preventing direct contact between MoRe and the edge of the mesa. (**B**) Schematic side view of a vertical cross section of (A). (**C**) Resistance map as a function of back-gate voltage, *V*_BG_, and symmetrically applied side-gate voltages, *V*_SG1_ = *V*_SG2_, at *B* = 1.8 T. The diamond-shaped regions correspond to the plateaus of quantized resistance. Their horizontal boundaries (affected by *V*_BG_ only) correspond to constant electron density in the bulk. The inclined side boundaries of the diamonds correspond to constant filling factors near the edges, where the influences of the back and the side gates compensate each other. The white numbers mark the sample’s filling factor, while the black numbers at the high side gate mark sample conductance in units of *e*^2^/*h*. (**D**) Finite element electrostatic simulation of (C) reproducing the diamond-shaped regions of constant conductance. The conductance plateaus marked in (C) are marked similarly. Computational details are provided in the Supplementary Materials. (**E**) Sample resistance as a function of *V*_BG_ at several *V*_SG1,2_, corresponding to vertical cross sections of (C). The curves show that the QH plateaus are best developed with the side gates set to −1 V. At *V*_SG1,2_ = − 3 V and +1 V, the plateaus shrink and become asymmetric between the electron and hole-doped sides, as is often found in samples without side gate control.

Our samples are made from graphene encapsulated in hexagonal BN, which protects devices from processing contamination and can yield ballistic transport over micrometer scales (*28*, *29*). The graphene-BN stack is then etched, and quasi-1D contacts to the exposed regions are fabricated (*30*). We use molybdenum rhenium (MoRe), a type II superconductor with an upper critical field of at least 9 T and critical temperature of ~9 K. The 3-μm-wide contacts are separated by 500 nm and are initially made to an extended region of graphene. At the next stage, both the junction and the side gates are formed by etching narrow trenches on each side of the contacts (Fig. 1A). Applying voltage to the graphene regions that form the side gates allows us to efficiently control the electron density along the edges of the junction (*31*, *32*). It is important that the etched trenches do not overlap with the contacts and are instead spaced from them by a graphene strip ~100 nm wide. This strip separates the contacts from any atomic-scale spurious states that may exist along the etched edges, precluding the possibility of electrons tunneling directly from the superconductor to the edge. For consistency, we present results from one Josephson junction; additional measurements of a second device are shown in the Supplementary Materials.

## RESULTS

### Side gate influence in the QH regime

**Fig. 2:**
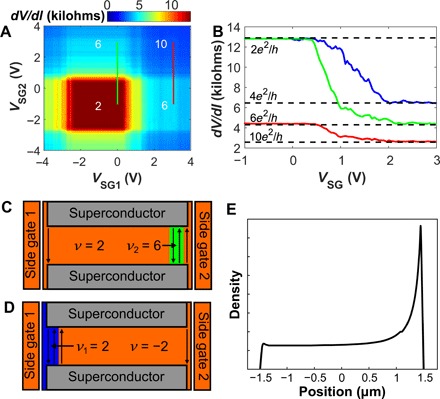
Side gate–induced QH plateaus. (**A**) *dV*/*dI* map plotted versus side-gate voltages *V*_SG1_ and *V*_SG2_ at *B* = 1.8 T. The back-gate voltage is fixed at *V*_BG_ = 4.7 V, corresponding to the bulk ν = 2 state. The numbers mark the sample conductance in units of *e*^2^/*h*. (**B**) Sample resistance measured as a function of a single side gate. Green and red curves correspond to the vertical lines in (A) at *V*_SG1_ = 0 and 3 V, respectively (with *V*_BG_ = 4.7 V). The blue curve shows a similar trace with a bulk filling factor ν = − 2 (*V*_BG_ = 1.5 V), sweeping *V*_SG1_ with *V*_SG2_ = 0 V. (**C** and **D**) Schematics corresponding to the green and blue curves in (B) for *V*_SG_ greater than ∼2 V. Additional edge channels are created near the gate, with local filling factor ν_2_ = 6 (C, green region) and ν_1_ = 2 (D, blue region). Additional conductance is equal to 4*e*^2^/*h* and 2*e*^2^/*h* in (C) and (D), respectively, on top of the base conductance of 2*e*^2^/*h*, as is observed for the blue and green curves in (B). (**E**) Schematic of the carrier density within the graphene junction as a function of position when SG2 (1) is active (passive), akin to (C).

**Fig. 3:**
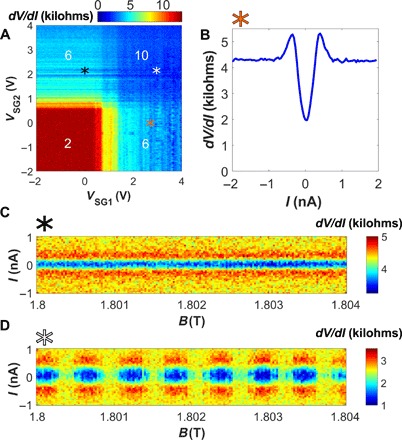
QH supercurrent and its interference patterns. (**A**) Differential resistance map versus *V*_SG1,2_ as in Fig. 2A but measured with 0 nA DC current bias, allowing observation of suppressed resistance due to the supercurrent. The gate voltage locations of (**B**) to (**D**) are marked by (B) an orange asterisk, (C) a black asterisk, and (D) a white asterisk. (B) *dV*/*dI* measured versus *I* indicating the presence of a supercurrent on top of the quantized *h*/6*e*^2^ plateau. (C) Current-magnetic field map of the differential resistance when a supercurrent is induced along one side of the sample only with *V*_SG2_, while *V*_SG1_ stays at zero. The supercurrent is not sensitive to an incremental change of field on a few millitesla scale. (D) A similar map with both side gates inducing supercurrent, showing a SQUID-like interference pattern.

As a magnetic field *B* is applied perpendicular to the sample, the junction enters the QH regime. By 1.8 T, the QH effect is very well developed, and we stay at that field in Figs. 1 to 3. The influence of the side gates is substantial in this regime, since the edges of the device dominate the transport properties. Figure 1C maps the influence of the back gate and the two side gates, applied symmetrically, *V*_SG1_ = *V*_SG2_. This and subsequent measurements in this section are performed with a DC (direct current) bias of 10 nA, enough to suppress any supercurrent that may be flowing between the contacts in the QH regime. An additional, negligibly small alternating current (AC) of 50 pA is applied to measure the differential resistance with a lock-in amplifier. The large central red (high resistance) features in Fig. 1C mark the ν = ± 2 QH plateaus. Above and below these are the standard ν = ± 6 states. Only the $\nu =4(n+\frac{1}{2})$ sequence of filling factors is visible at this field.

The regions of quantized conductance have a diamond shape, whose boundaries in the back gate direction are flat (horizontal), which means that they are not affected by the side gates. The inclined side boundaries of the red diamonds indicate that they depend both on the side gates and the back gate. These boundaries are interpreted as a line of constant carrier density along the edges of the device, *n*_side_ ∝ (*V*_SG1,2_ − α*V*_BG_) = const, where α ∼ 2 is a constant determined by the relative gate efficiencies. The overall shape of the map in Fig. 1C is well reproduced by a simple electrostatic simulation, as shown in Fig. 1D.

Last, the centers of the diamond-shaped plateaus in Fig. 1C are shifted from *V*_SG1,2_ = 0 V, indicating that the “neutral” side-gate voltage is close to −1 V. This differs from the back-gate position of the charge neutrality point (3.5 V) not only in magnitude but also in polarity, indicating a carrier buildup along the edges of the junction distinct from the doping of the bulk. The side gate influence is illustrated in Fig. 1E, which demonstrates that the resistance plateaus of the device, as a function of the back gate, are better formed at *V*_SG1,2_ = − 1 V than at −3 or +1 V.

More insight into the device’s phenomenology is gained by applying the side gates independently. Figure 2A shows a resistance map of the device as a function of both side gates at *V*_BG_= 4.7 V. (Taking a *V*_SG1_ = *V*_SG2_ diagonal line in Fig. 2A would correspond to a horizontal line going through the middle of the ν = 2 diamond in Fig. 1C.) The prominent feature of Fig. 2A is a square central region with resistance quantized at *R* = *h*/2*e*^2^. When either side gate is applied beyond the plateau region, the resistance drops to a different quantized value.

The observed influence of the side gates on the QH conductances is similar to the impact of local out-of-plane gates (*33*, *34*). The fact that the features in Fig. 2A are purely horizontal or vertical shows that the influence of the two side gates is highly local: The left gate has a negligible effect on the right edge and vice versa. This negligible cross-talk is different from that typically found in samples with out-of-plane gates. Furthermore, the side gates are efficient and tune the local density by ~10^11^ cm^−2^ per volt, compared with ~7 × 10^10^ cm^−2^ per volt for the back gate. In particular, we are able to change the filling factor along either edge.

Figure 2B shows that the measured resistance drops from *R* = *h*/2*e*^2^ to *R* = *h*/6*e*^2^, if a positive side-gate voltage is applied (green curve, measured along the green line in Fig. 2A). This corresponds to ν_2_, the local filling factor on the side close to side gate 2 (SG2), reaching ν_2_ = 6 as shown schematically in Fig. 2C. The bulk filling factor remains at ν = 2, and an additional conductance of 4*e*^2^/*h* is contributed by the additional fourfold degenerate edge states induced near SG2. Note that in this case, the spatial separation between counter-propagating QH states in the side-gated region is less than 100 nm, as detailed further in the text. The observation of quantized resistance plateaus suggests that backscattering between these counter-propagating states is suppressed, despite their close proximity. Indeed, robust QH plateaus were previously observed in graphene nanoribbons of comparable width (*35*, *36*).

Next, the red line of Fig. 2B demonstrates that each side gate can induce an independent ν = 6 state along its edge. Here, SG1 is fixed at 3 V; this corresponds to a local filling factor near SG1 of ν_1_ = 6. Before SG2 is applied, we start with resistance of *h*/6*e*^2^: The baseline conductance is 2*e*^2^/*h*, and the right edge contributes additional 4*e*^2^/*h*, much like at the end point of the green curve in Fig. 2B. Applying SG2 then adds an additional fourfold degenerate channel on the other edge of the sample, resulting in the drop of resistance to *h*/10*e*^2^, which corresponds to conductance of (2 + 4 + 4)*e*^2^/*h*.

Last, we tune the back gate to 1.5 V (instead of 4.7 V), resulting in a bulk filling of ν = − 2. Applying SG1 now yields a transition from *R* = *h*/2*e*^2^ to *R* = *h*/4*e*^2^ (blue curve in Fig. 2B.) The schematics in Fig. 2D shows that in this case, the side gate locally induces a QH state with an opposite filling factor of ν = 2, and the resulting plateau has a conductance of (2 + 2)*e*^2^/*h*. Note that here as well, counter-propagating states are created in close proximity to each other.

### Side gates and QH supercurrent

So far, the measurements have been performed with an applied DC bias current *I* of 10 nA to suppress any supercurrent. We now switch *I* to zero and explore the emerging superconducting features, maintaining the small AC current of 50 pA used to measure the differential resistance. Figure 3A shows a map of sample resistance versus side gates similar to that in Fig. 2A. While no supercurrent is found on top of the ν = 2 plateau, once the ν = 6 state is induced by either side gate, the sample resistance develops pronounced dips that were not present at high DC current.

Figure 3B shows the sample resistance versus bias taken at the location in Fig. 3A marked by an orange asterisk, corresponding to *V*_SG2_ = 0 V and *V*_SG1_ = 2.5 V, so that ν_2_ is close to bulk filling and ν_1_ = 6. The region of suppressed resistance flanked by peaks is characteristic of a small supercurrent washed by thermal fluctuations. Notice that when the density enhancement is induced on one side only (regions in Fig. 3A corresponding to the normal resistance of *h*/6*e*^2^), the supercurrent features appear as horizontal/vertical lines—they depend on one side gate and do not vary with the other side gate. This confirms that the supercurrent is localized at one side of the junction.

Furthermore, the supercurrent does not vary for small changes in magnetic field (Fig. 3C), indicating that the area it encompasses does not enclose additional flux quanta for a few millitesla change in field. This observation limits the distance between the counter-propagating edge channels responsible for the supercurrent to no more than ∼100 nm (see also fig. S1C). This distance is comparable to the coherence length of MoRe, which facilitates the coupling of the edge states to the superconductor and explains the appearance of a supercurrent when a side gate is turned on.

The dependence of the supercurrent on magnetic field completely changes when both side gates are applied, creating supercurrents along the two edges of the sample. Figure 3D shows a map similar to Fig. 3C, but with both side gates applied (*V*_SG1_ = 3.04 V, *V*_SG2_ = 2.11 V, marked by a white asterisk in Fig. 3A). The map demonstrates a superconducting quantum interference device (SQUID)–like interference pattern with a period of 0.6 mT, close to that of the low-field Fraunhofer pattern of this junction (0.7 mT).

**Fig. 4:**
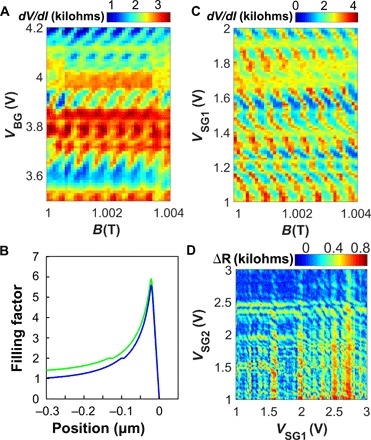
QH supercurrent interferometry. (**A**) *dV*/*dI* map measured at *V*_SG1_ = 2.34 V and *V*_SG2_ = 2.36 V as a function of *V*_BG_ and *B* near 1 T. For a given gate voltage, the regions of suppressed resistance correspond to stronger supercurrent. The pattern is periodic in *B* with the same period as in Fig. 3D. The phase of the oscillations depends on the gate voltage, indicating that the interference area decreases with the gate voltage (positive *dV*_BG_/*dB*). This is explained by the inner edge states moving further inward as the electron density grows [schematic in (B)]. (**B**) Schematic of carrier density in the sample along the midline between the contacts. The blue line represents some baseline charge density; the green line shows a higher back gate voltage. These curves are generated using the electrostatic model discussed in the Supplementary Materials, but here are meant to be qualitative. (**C**) *dV*/*dI* map similar to (A) measured as a function of *B* and SG1 voltage for *V*_BG_ = 3.8 V. The map shows an interference pattern with a slope opposite that in (A), indicating that the interference area increases with gate voltage as the electrons are pushed further toward the gate. (**D**) ΔR map displaying the difference between the resistance in the 0 and 10 nA DC bias conditions, measured at 1 T with *V*_BG_ = 3.9 V. Both side-gate voltages are high enough to induce a supercurrent (*V*_SG1,2_ > 1 V), and the vertical and horizontal features correspond to the supercurrent induced by SG1 or SG2, respectively. At their intersections, additional diagonal features appear, indicating interference between the supercurrents on the two sides of the sample. The fringes have a slope ∼ −1, suggesting comparable efficiency of the two side gates.

We explore the device as an interferometer for QH supercurrents in Fig. 4. Here, we change the field to 1 T to observe a more robust superconducting signature. Figure 4A shows the pattern of resistance oscillations in magnetic field, measured at zero applied DC bias as a function of the back gate. The period of the oscillations is found to be the same as in Fig. 3D and independent of the gate voltage. The phase of the oscillations, however, is seen to vary with gate with an approximate slope of +150*V*_BG_/*T*.

This gradual shift of the magnetic interference pattern with the back gate is explained by the fact that the changing electron density shifts the position of the QH edge states, thereby changing the area between the supercurrents on the two sides. The phase change from an increase in density (at more positive *V*_BG_) is compensated for by the increase in the magnetic field, indicating that the effective area of the SQUID shrinks. This behavior can be understood from the schematic in Fig. 4B, where the blue curve (lower back-gate voltage) is compared to the green line (higher back-gate voltage). The counter-propagating edge states occur on the opposite slopes of the nonmonotonic density profile close to each edge. As the overall density increases (from the blue to the green curve), the inner states move further inward, while the outer states stay relatively stationary due to the very high density gradient close to the sample edge. As a result, on average, the location of the supercurrent moves inward with increasing density.

A similar change in the interference pattern is observed when a side gate is applied (Fig. 4C). The slope of this pattern is roughly −300*V*_SG1_/*T*. Notably, the sign of the slope in Fig. 4C is flipped compared with the one seen in Fig. 4A. Following the discussion in the previous paragraph, this slope suggests that applying the side gate may be increasing the effective area of the SQUID. This could likely be attributed to the outward shift of the outer edge state, which is more strongly influenced by the side gate than the inner edge state. The very small size of the graphene region affected by the side gate might also result in charging effects, which are known to invert the slope of fringes in QH interferometers (*37*–*39*).

Last, an additional interference pattern is revealed in Fig. 4D, which shows the ΔR, the difference in the sample resistance between 0 and 10 nA DC bias, which highlights the superconducting regions. The map is measured as a function of both side gates at *B* = 1 T and *V*_BG_ = 3.9 V. The interference is visible at the intersections of the vertical and horizontal lines corresponding to supercurrents flowing along the SG1 and SG2 edges, respectively. The interpretation of this interference pattern is similar to the discussion above, with each gate affecting the location of the edge state on its side of the device: The gates change the phase by inducing small changes of the area of the SQUID. Lines of the constant phase correspond to the situation in which the area reduction on one side is compensated by the area increase on the opposite side, so that the total area stays constant. The contours of the constant phase at the intersections of the vertical and horizontal lines have a roughly diagonal slope, indicating that the two gates have comparable efficiency—as one gate voltage is increased; decreasing the opposite gate voltage accordingly maintains roughly the same area of the SQUID. Note that these area changes are sufficient to evolve the phase difference across the junction, but too small to create noticeable changes in the magnetic field periodicity.

## DISCUSSION

We have shown that native graphene side gates are remarkably efficient in controlling edge state propagation in the QH regime. They enable full control of the local filling factors along the sample edges, allowing us to fill the next Landau level, change carrier polarity, or keep the density flat close to the edge. Furthermore, we have observed supercurrents carried by the QH edge states induced by the side gates. These supercurrents flow independently on each edge of the device and could be controlled independently by the corresponding gates. Our experiment opens a promising route for coupling superconductors with QH edge states for the purpose of inducing non-Abelian excitations.

## MATERIALS AND METHODS

The sample was made with mechanically exfoliated flakes of graphene and hexagonal BN. It was assembled using a standard stamping technique (*30*). The resulting heterostructure was patterned using electron beam lithography followed by reactive ion etching with CHF_3_ and O_2_ to expose the edges of the encapsulated graphene. These edges were contacted with 100 nm of MoRe (50:50 ratio by weight) sputtered onto the etched regions. The device boundaries and side gates were defined with a second round of lithography and etching.

Measurements were performed in a Leiden Cryogenics dilution refrigerator at a temperature of ~100 mK. The sample was electronically isolated in the refrigerator via resistive coax lines and low-temperature RC filters. Differential resistance measurements were carried out using an AC excitation current of 50 pA. Magnetic fields for QH measurements were applied perpendicular to the sample plane.

## Supplementary Material

http://advances.sciencemag.org/cgi/content/full/5/9/eaaw8693/DC1

Download PDF

Quantum Hall–based superconducting interference device

## SUPPLEMENTARY MATERIALS

Supplementary material for this article is available at http://advances.sciencemag.org/cgi/content/full/5/9/eaaw8693/DC1 Section S1. Additional supercurrent interference maps Section S2. Measurements around the bulk ν = 6 plateau Section S3. Measurements of a second device Section S4. Electrostatic simulations Section S5. Magnetic interference patterns Fig. S1. Additional side gate maps and interference patterns at 1.8 T. Fig. S2. Additional side gate maps and interference patterns at 1 T. Fig. S3. Supercurrent at ν = 6 in the bulk at 1 T. Fig. S4. Study of a second device at 1 T. Fig. S5. Simulated evolution of carrier density near the junction edge. Fig. S6. Three column comparison of the supercurrent distributions and the resulting magnetic interference patterns.
