# Supplementary material for: Quantum Hall–based superconducting interference device
Source: Sci Adv. 2019 Sep 13;5(9):eaaw8693. doi: 10.1126/sciadv.aaw8693 (PMC6744260; doi:10.1126/sciadv.aaw8693)
Supplement: http://advances.sciencemag.org/cgi/content/full/5/9/eaaw8693/DC1 [file supp_5_9_eaaw8693__index.html]

Science Advances | Science AdvancesAAASSearchScience AdvancesMenu

## Supplementary Materials

**This PDF file includes:**

- Section S1. Additional supercurrent interference maps
- Section S2. Measurements around the bulk ν = 6 plateau
- Section S3. Measurements of a second device
- Section S4. Electrostatic simulations
- Section S5. Magnetic interference patterns
- Fig. S1. Additional side gate maps and interference patterns at 1.8 T.
- Fig. S2. Additional side gate maps and interference patterns at 1 T.
- Fig. S3. Supercurrent at ν = 6 in the bulk at 1 T.
- Fig. S4. Study of a second device at 1 T.
- Fig. S5. Simulated evolution of carrier density near the junction edge.
- Fig. S6. Three column comparison of the supercurrent distributions and the resulting magnetic interference patterns.

Download PDF

**Files in this Data Supplement:**

- Adobe PDF - aaw8693\_SM.pdf
