# Supplementary material for: Quantum Hall–based superconducting interference device
Source: Sci Adv. 2019 Sep 13;5(9):eaaw8693. doi: 10.1126/sciadv.aaw8693 (PMC6744260; doi:10.1126/sciadv.aaw8693)
Supplement: Download PDF [file aaw8693_SM.pdf]

Science Advances
AAAS

advances.sciencemag.org/cgi/content/full/5/9/eaaw8693/DC1

Supplementary Materials for

Quantum Hall–based superconducting interference device

Andrew Seredinski*, Anne W. Draelos, Ethan G. Arnault, Ming-Tso Wei, Hengming Li, Tate Fleming, Kenji Watanabe, Takashi Taniguchi, François Amet, Gleb Finkelstein

*Corresponding author. Email: ams168@duke.edu

Published 13 September 2019, *Sci. Adv.* **5**, eaaw8693 (2019)
DOI: 10.1126/sciadv.aaw8693

This PDF file includes:

Section S1. Additional supercurrent interference maps
Section S2. Measurements around the bulk ν = 6 plateau
Section S3. Measurements of a second device
Section S4. Electrostatic simulations
Section S5. Magnetic interference patterns
Fig. S1. Additional side gate maps and interference patterns at 1.8 T.
Fig. S2. Additional side gate maps and interference patterns at 1 T.
Fig. S3. Supercurrent at ν = 6 in the bulk at 1 T.
Fig. S4. Study of a second device at 1 T.
Fig. S5. Simulated evolution of carrier density near the junction edge.
Fig. S6. Three column comparison of the supercurrent distributions and the resulting magnetic interference patterns.

Supplementary material

Section S1. Additional supercurrent interference maps

Figure S1 expands on the 1.8 T data presented in Figures 1-3 in the main text. Panel a shows a wider version of the SG1-SG2 map of differential resistance seen in Figure 3a with $I_{DC} = 0$ nA. Here, in addition to the side gate induced quantum Hall plateaus, we see lines of reduced resistance as supercurrent appears at certain side gate voltages on either side of the junction. The panels of Figure S1b show the magnetic interference pattern of one supercurrent pocket induced by SG2 as SG1 changes from 0 to +3 V, following the dashed line in Figure S1a. Once both gates are ≥ 2 V, the supercurrents along the two edges interfere and we see the transition from an aperiodic to a fully SQUID-like interference pattern. Finally, Figure S1c shows aperiodic resistance measured as a function of magnetic field and SG1, with SG2 held at 0 V. This contrasts with the periodic side gate - field interference map taken in the regime when both edges carry supercurrent (Figure 4c). Clearly, since the supercurrent is independently controlled along each edge, only when both edges are active is there interference. As stated in the main text, we interpret the interference observed in Figure 4d as the result of each side gate tuning the location of its associated edge states, thus altering the enclosed magnetic flux through the junction.

Figure S2 presents additional interference data at $B = 1$ T. S2a and b contrast the differential resistance maps taken at $I_{DC} = 10$ nA and $I_{DC} = 0nA$; S2b is an extended version of the map in Figure 4d. It is clear that the map in S2a lacks the diagonal interference features present in S2b. Indeed, these features are attributed to interference of supercurrents, which are suppressed by $I_{DC} = 10$ nA. Figures S2c,d detail the transition of the side gate interference pattern from periodic to aperiodic as a function of each side gate. This is consistent with the picture of independent supercurrents on each edge of the device being present at only certain local gate voltages.

Section S2. Measurements around the bulk ν =6 plateau

The main text focuses on data taken when the bulk of the sample is tuned to the $\nu = 2$ plateau. In this section, we include complementary data taken at the $\nu = 6$ plateau in the bulk, at $B = 1$ T. In Figure S3a, increasing either side gate leads to the total differential resistance reaching $h/10e^2$. This indicates the addition of a fourfold degenerate state only along one side of the junction. Tuning the second gate, understandably, yields $h/14e^2$: Each side of the junction can be thought of as locally being in the $\nu = 10$ state, while the bulk remains at $\nu = 6$. Each edge contributes $4e^2/h$ in addition to the base $6e^2/h$ conductance. Figure S3b presents a map of differential resistance at $I_{DC} = 0$nA, showing a multitude of locations with superconducting pockets.

Section S3. Measurements of a second device

In this section we present measurements similar to those in the main paper, but taken on a different device (J2). The dimensions of J2 are similar to J1, and the junction region is also separated from the two side gates by ~ 60 nm - wide trenches. However, here the contacts are not spaced from the trenches by 100 nm regions of graphene, as was done in J1. This second device was fabricated on the same chip as the main device. In fact, SG2 is shared by both junctions (Figure S4a).

The data presented were taken at $B = 1$ T with $V_{BG} = 1.85$ V in the center of the $\nu = -2$ plateau. Side gate - side gate maps of differential resistance are presented in Figure S4b,c and show the development of new resistance plateaus at high side gate voltage. These correspond to $h/4e^2$ when one side gate is applied, and to $h/6e^2$ when both are applied. These values are explained by the appearance of $\nu = 2$ channels along each side of the junction. (See Figure 2d in the main text.)

Like our main junction J1, this device shows regions of supercurrent in the $I_{DC} = 0$ nA bias map (Figure S4c). As seen previously in Figure S1d, the dependence of supercurrent on magnetic field also undergoes a transition from non-periodic to SQUID-like: compare Figure S4d, in which only SG2 gate is active, to Figure S4e, in which SG3 is also applied, resulting in interference.

Section S4. Electrostatic simulations

In order to simulate the carrier density profile induced by the side gate in the quantum Hall regime, we determined the geometric local capacitance between the graphene sheet and both the back gate and side gates.

We solve the Laplace equation for the electrostatic potential with Dirichlet boundary conditions at the back gate, side gates, and the graphene sheet. Both local capacitances are spatially varying and are stronger at the graphene edge as a result of electric field focusing.

The back gate and the intrinsic doping n_0 of the graphene sheet (determined experimentally by the location of the Dirac peak in a gate sweep of the device) were then used to determine the bulk carrier density in the graphene and subsequently the chemical potential.

Applying a positive voltage on the side gate tends to raise the graphene potential near the edge. At zero temperature the local graphene potential $V_g(x)$ can be obtained by solving

$$n_0 + C_{BG}(x)(V_{BG} - V_g(x)) + C_{SG}(x)(V_{SG} - V_g(x)) = \int_0^{\mu+V_g} \rho(E)dE \tag{S1}$$

Here, we defined the density of states as a sum of Gaussians centered at the Landau level energies E_n with a Landau level degeneracy N

$$\rho(E) = N\sum_{n=0}^{\infty} \frac{1}{\tau\sqrt{2\pi}} exp(-\frac{1}{2}(\frac{E-E_n}{\tau})^2) \tag{S2}$$

where τ parametrizes the breadth of the Landau levels. The local carrier density is then

$$n(x) = \int_0^{\mu+V_g} \rho(E)dE \tag{S3}$$

The vanishing of the carrier density at the edge was artificially obtained by linearly bringing the density to zero at the edge over a length scale l. The length scale of the convergence was chosen to be on the order of the magnetic length ($\sim$ 20nm) and justified post hoc through comparison of the simulated and experimental gate maps. This behavior is of course a simplification and further theoretical work will be needed to understand the evolution of the carrier density in the few nanometers near the edge.

Figure S5 details the evolution of carrier density within 300nm of the junction edge with the application of back gate (Figure S5a) and side gate (Figure S5b) voltages. Figure S5a quantitatively builds on Figure 4b from the main text. We note that the electrostatic simulation of the side gate influence in Figure S5b does not reproduce the movement of the supercurrent carrying states towards the edge of the junction, as is discussed in the main text in explaining the slope of the interference pattern shown in Figure 4c.

Finally, to reproduce the differential resistance map shown in Figure 1c, the carrier density was used to determine the number of edge states in the system and plotted as a function of gate, generating Figure 1d. Commensurate with the integer plateaus seen in the experiment, we considered only the degenerate graphene filling factors ($\nu = 2, 6, 10, \dots$) and added in the emerging $\nu = 0$ peak (defined in the model for densities corresponding to $n_{\nu=-1} < n < n_{\nu=1}$), which was assigned an arbitrary conductance for map contrast (dark red in the map of Figure 1d).

Section S5. Magnetic interference patterns

The periodic variation of supercurrent as a function of the magnetic field (as explored in Figures 3 and 4 of the main text) is a consequence of the magnetic flux penetrating the area of the sample. Figure S6 schematically shows the distribution of the supercurrent across the junctions in different regimes. We contrast the uniform distribution at zero field and the supercurrent carried along one or both edges in the quantum Hall regime.

Notice that when both side gates are applied, two supercurrent paths are formed; their interference in magnetic field results in SQUID-like oscillations. This does not happen when only one side gate is applied, resulting in a single superconducting path.

Regarding the SQUID oscillations observed in Figure 4, note that a small gate-induced shift of the QH edge states by just a few nanometers would be sufficient to change the interference phase by 2π. At the same time, such a change is orders of magnitude too small to noticeably impact the period of oscillations. Indeed, to change the observed period (0.6-0.7 mT) by 0.1 mT (our resolution), the area would have to change by about 15%, which means that the edge states would have to shift by hundreds of nanometers.

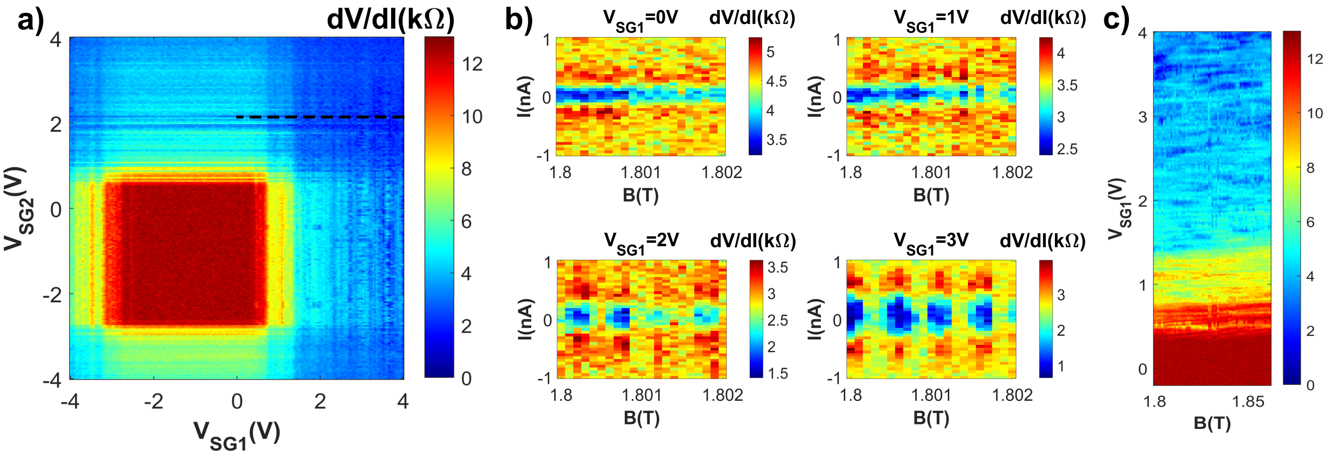

Fig. S1. Additional side gate maps and interference patterns at 1.8 T. a) Differential resistance map measured as a function of both side gates at $I_{DC} = 0$ nA and bulk filling $\nu = 2$. This is an extended version of the map shown in Figure 3a of the main text. Large enough negative side gate voltages result in transitions from the central $\nu = 2$ plateau to the regions of differential resistance equal to $h/4e^2$ (as explained in Figure 2d). Supercurrent pockets again appear at certain side gate values as vertical or horizontal lines of suppressed resistance. b) Evolution of the supercurrent vs. B maps as SG1 grows from 0 to 3 V along the dashed line in (a). The maps show the transition from aperiodic supercurrent at $V_{SG1} = 0$ V to a SQUID-like periodicity at $V_{SG1} = 3$ V. This illustrates the change of behavior from supercurrent on one side of the junction to supercurrent on both sides. c) Map of differential resistance as a function of SG1 and magnetic field, taken at 0 DC bias. This is similar to Figure 4c, but here $V_{SG2} = 0$ V, so supercurrent is induced only along SG1. The supercurrent is clearly aperiodic, showing only some irregular dependence on magnetic field. Note that compared to Figure 4a,c of the main text, the range of fields here is about 10 times wider.

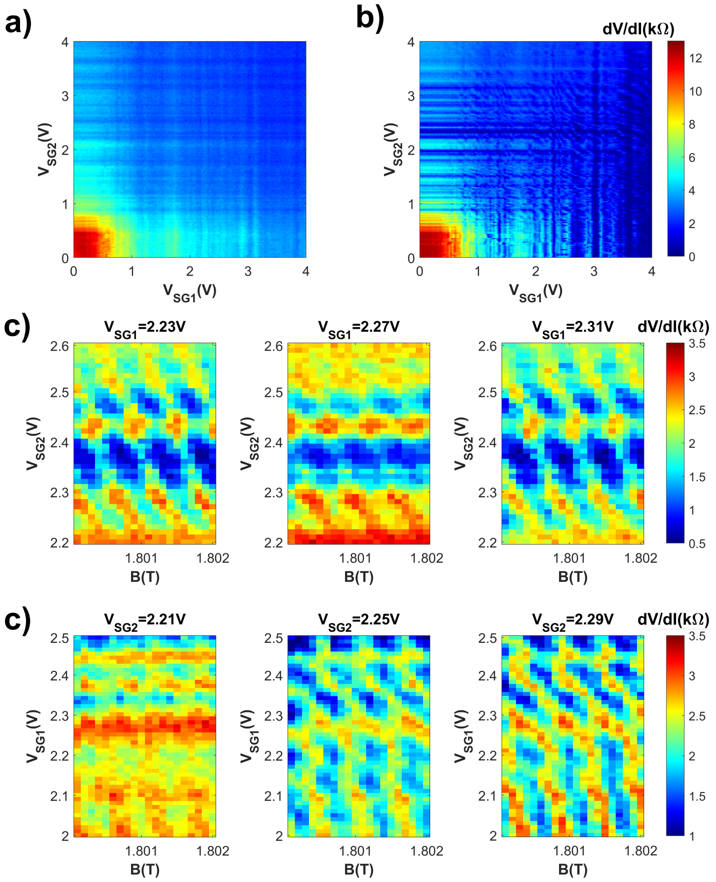

Fig. S2. Additional side gate maps and interference patterns at 1 T. a-b) Wider SG1-SG2 maps of differential resistance at a) 10 nA and b) 0 nA DC bias. The map in (a) lacks the diagonal interference features of (b), which are interpreted as a supercurrent interference effect and so are not present in the 10 nA bias condition. c) Maps of differential resistance measured as a function of SG2 and field B at 0 DC bias. The maps are taken at three SG1 locations (2.23, 2.27, 2.31 V) in the regime when the two supercurrents interfere. The observed patterns are very similar, except for the shift of the phase of the oscillations and the overall contrast. These changes are explained by the change in the strength and geometrical position of the supercurrent flowing along along SG1 as the corresponding voltage is changed between the three maps. Note that at the bottom of all maps ($V_{SG2} = 2.2$ V) the pattern becomes roughly independent of magnetic field, because at that value of SG2 the corresponding current is equal to zero. d) Similar to (c), but here SG1 is swept and SG2 is fixed at 2.21, 2.25, and 2.29 V. Note that the left map taken at $V_{SG2} = 2.21$ V is barely periodic because the current flowing along SG2 is very small.

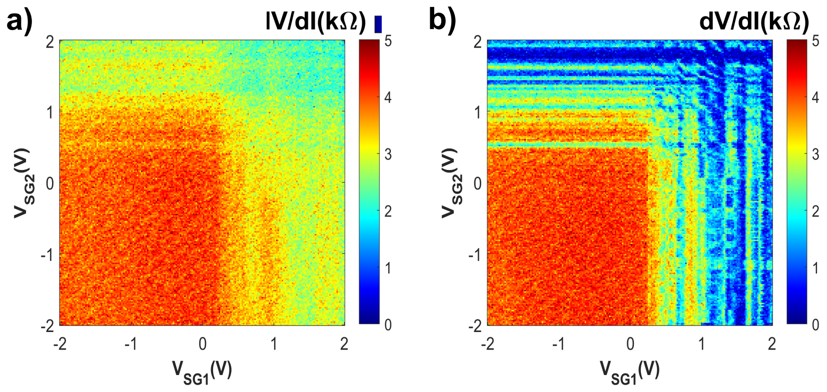

Fig. S3. Supercurrent at ν = 6 in the bulk at 1 T. a-b) SG1-SG2 maps of differential resistance taken at back gate voltage $V_{BG} = 5.55$ V, which corresponds to $\nu = 6$ filling in the bulk. The maps are measured at a) 10nA and b) zero DC bias. In a) the plateaus have resistances of $h/6e^2$ (side gate equal to zero or negative), $h/10e^2$ (one side gate applied), and $h/14e^2$ (both side gates on). The SG1-SG2 interference effect shown in Figure 4e is also seen here in panel (b).

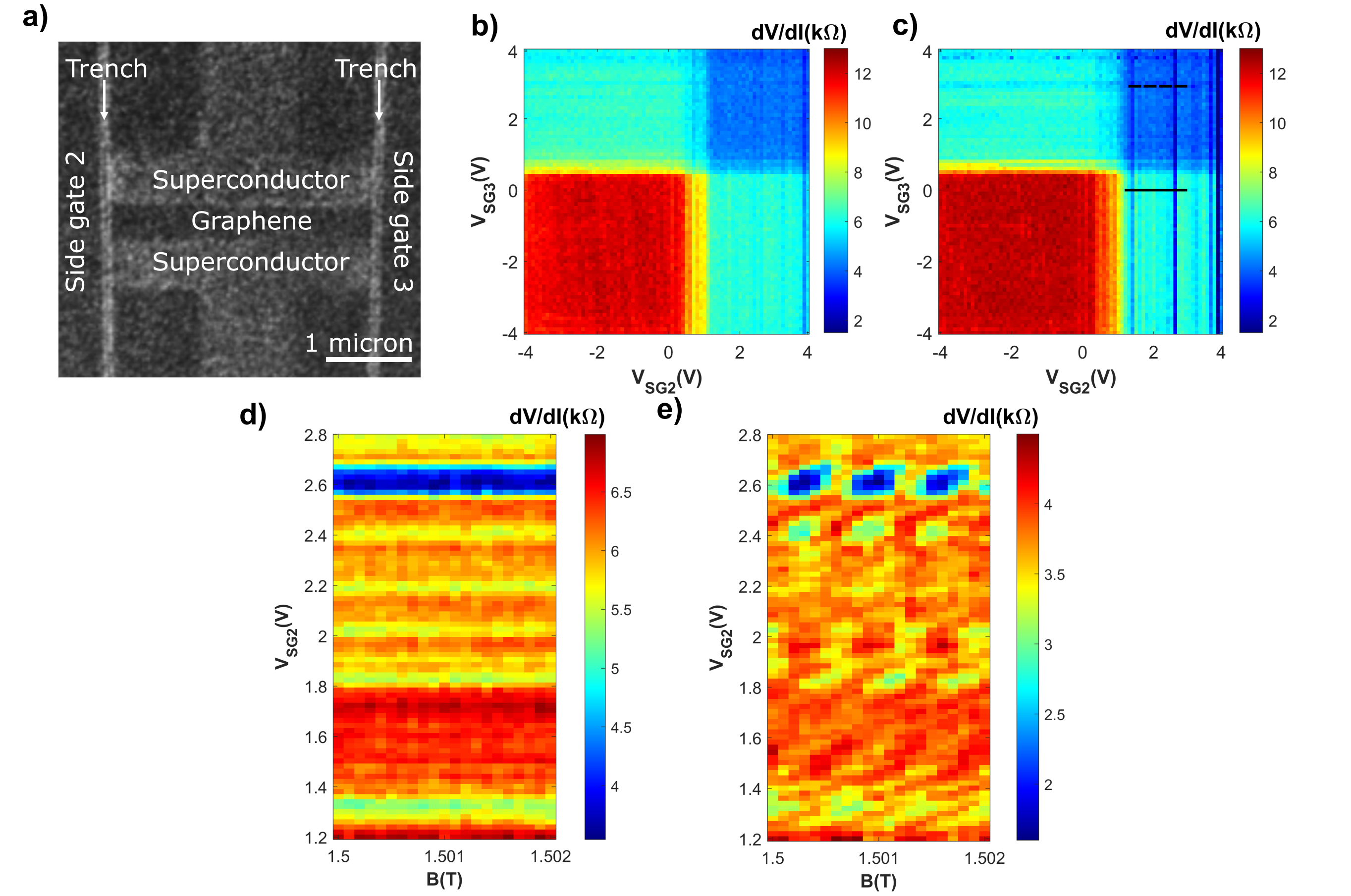

Fig. S4. Study of a second device at 1 T. a) SEM micrograph of the device prior to reactive ion etching. MoRe contacts are again visible in lighter gray. Two trenches, $\sim$ 60 nm wide, separate the junction from the side gates. The side gates are labeled SG2 and SG3, where SG2 is shared with the device from the main paper. b-c) SG2-SG3 maps of differential resistance taken at b) 10nA and c) 0na DC bias like in the previous figures. The maps are taken at $V_{BG} = 1.85$ V, corresponding to $\nu = -2$. Panel (b) shows that one side gate induces a resistance of $h/4e^2$, and both side gates induce a resistance of $h/6e^2$, as expected from the study of the first device. Panel (c) shows the development of supercurrent at positive SG voltages. The solid and dotted lines show the location of the maps in (d) and (e), respectively. d) Map of differential resistance as a function of SG2 and field at $V_{SG3} = 0$ V. Aperiodic supercurrent is seen (e.g. see the pockets at V_{SG2} =1.3 and 2.6 V), which again indicates the localization of the supercurrent along one edge. e) Similar map of SG2 vs. field at $V_{SG3} = 2.9$ V showing a periodic supercurrent pattern that varies with gate. The sloped features show that the phase of the oscillations depends on gate voltage, indicating that the interference area changes with gate voltage, similar to the result of Figure 4c of the main text.

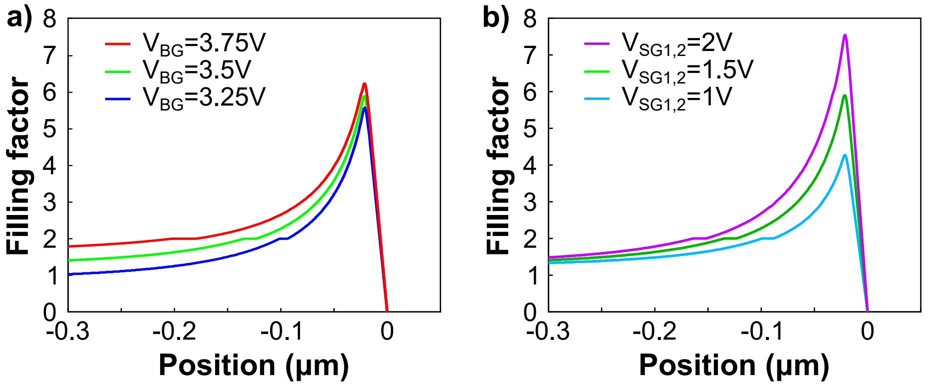

Fig. S5. Simulated evolution of carrier density near the junction edge. a) Simulated carrier density at several back gate locations (blue 3.25 V, green 3.5 V, red 3.75 V) within 300 nm of the sample edge with both side gates held at 1.5 V. This is as Figure 4c of the main text with the addition of the red curve and gate values given for quantitative comparison. Increasing the gate voltage is seen to impact the edge density somewhat and to have a strong impact on the bulk density away from the edge. The flat features are incompressible strips resulting from the quantum Hall effect. b) As (a) with $V_{BG} = 3.5$ V, but with the side gate location tuned (cyan 1 V, green 1.5 V, purple 2 V). The green curves on both (a) and (b) are identical. The side gate voltage is seen to significantly shift the edge density with only a modest impact even 300 nm into the bulk.

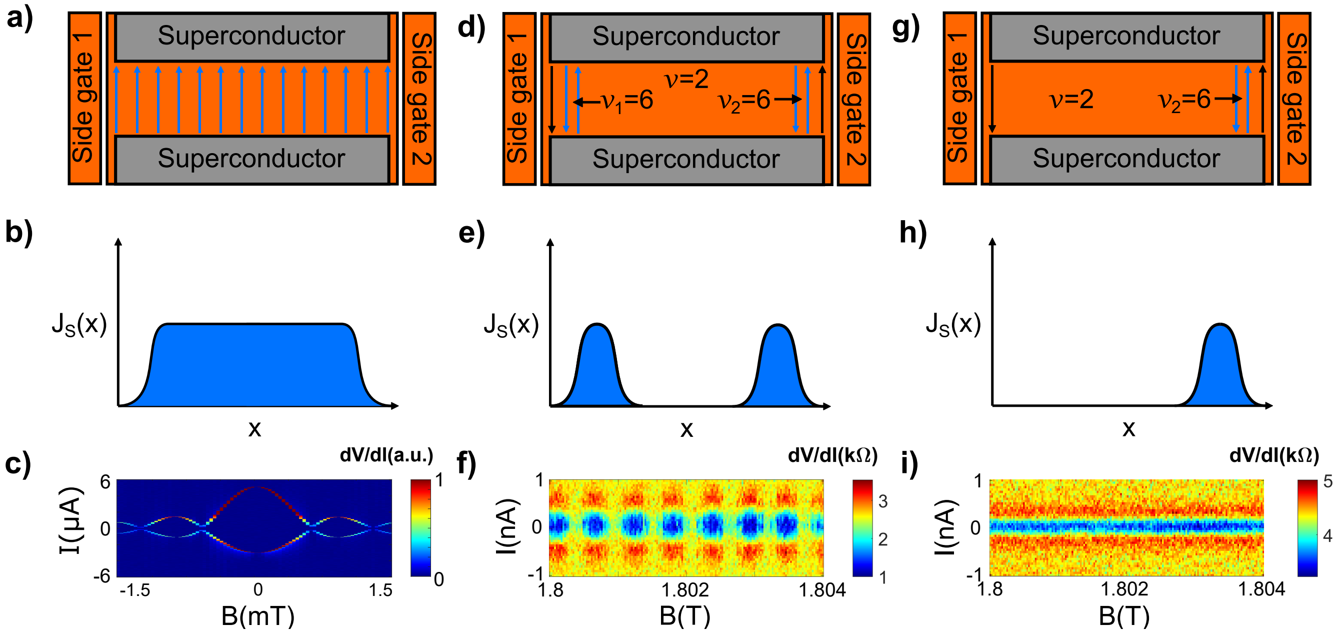

Fig. S6. Three column comparison of the supercurrent distributions and the resulting magnetic interference patterns. a) Diagram of supercurrent flow at low magnetic fields, resulting in a uniform distribution shown in panel (b). c) Measurement of the second device (see Figure S4a) at low magnetic field showing a typical Fraunhofer interference pattern with period of $\sim$ 0.7 mT, which indicates a uniform supercurrent distribution. Data is a numerical derivative of the measured I-V curves (arbitrary units). T= 230 mK. d) Schematic of QH edges when both side gates are applied (bulk $\nu = 2$, locally induced $\nu = 6$ on each edge). The closely spaced counterpropagating states support supercurrents on both edges of the sample, resulting in the distribution shown in panel (e). Panel (f) is reproduced from Figure 3d and shows the SQUID-like magnetic interference pattern of quantum Hall supercurrent corresponding to panel (d) with a periodicity of $\sim$ 0.6 mT. Panel (g) is similar to panel (d) but with only one side gate applied. This results in the supercurrent flowing only on one edge of the sample, as shown in panel (h). Panel (i) is reproduced from Figure 3c and shows the magnetic interference pattern corresponding to panel (g). There is no variation in the pattern on this field scale, indicating a single, highly localized distribution of current.
